# Differences in Diurnal Rhythm of Rod Outer Segment Renewal between 129T2/SvEmsJ and C57BL/6J Mice

**DOI:** 10.3390/ijms23169466

**Published:** 2022-08-22

**Authors:** Jade A. Vargas, Silvia C. Finnemann

**Affiliations:** Center for Cancer, Genetic Diseases and Gene Regulation, Department of Biological Sciences, Fordham University, Bronx, NY 10458, USA

**Keywords:** phosphatidylserine, phagocytosis, photoreceptors, RPE, mice

## Abstract

In all mammalian species tested to date, rod photoreceptor outer segment renewal is a circadian process synchronized by light with a burst of outer segment fragment (POS) shedding and POS phagocytosis by the adjacent retinal pigment epithelium (RPE) every morning at light onset. Recent reports show that RPE phagocytosis also increases shortly after dark onset in C57BL/6 (C57) mice. Genetic differences between C57 mice and 129T2/SvEmsJ (129) mice may affect regulation of outer segment renewal. Here, we used quantitative methods to directly compare outer segment renewal in C57 and 129 mouse retina. Quantification of rhodopsin-positive phagosomes in the RPE showed that in 129 mice, rod POS phagocytosis after light onset was significantly increased compared to C57 mice, but that 129 mice did not show a second peak after dark onset. Cone POS phagosome content of RPE cells did not differ by mouse strain with higher phagosome numbers after light than after dark. We further quantified externalization of the “eat me” signal phosphatidylserine by outer segment tips, which precedes POS phagocytosis. Live imaging of retina ex vivo showed that rod outer segments extended PS exposure in both strains but that frequency of outer segments with exposed PS after light onset was lower in C57 than in 129 retina. Taken together, 129 mice lacked a burst of rod outer segment renewal after dark onset. The increases in rod outer segment renewal after light and after dark onset in C57 mice were attenuated compared to the peak after light onset in 129 mice, suggesting an impairment in rhythmicity in C57 mice.

## 1. Introduction

In the vertebrate retina, the function of photoreceptor neurons for life is maintained through the process of photoreceptor outer segment renewal [1]. It involves the shedding of distal photoreceptor outer segment fragments (POS) and concomitant phagocytosis of POS by the adjacent retinal pigment epithelium (RPE) [1,2]. Photoreceptor outer segment renewal in mammals is a circadian and light-synchronized process [3]. In all mammalian species tested to date, rod POS shedding and phagocytosis occur in a burst shortly after light onset [3]. A robust morning peak shortly after light onset and low activity at other times is a rhythm that has been reported for over 50 years in laboratory mice and rats that are entrained to a 12 h light–12 h dark cycle, i.e., with light on from zeitgeber time 0 (ZT0) to ZT12 and light off from ZT12 to ZT0 [4,5,6]. Recently, a previously unseen evening peak of rod POS phagocytosis following onset of darkness has been reported in both mice and zebrafish [7,8,9]. In these studies, both electron microscopy and rhodopsin immunofluorescence microscopy were used to identify and quantify POS phagosomes in the RPE in situ. Lewis and colleagues found equivalent numbers of POS phagosomes in mouse RPE at ZT1.5 and at ZT13.5 [8]. Goyal and colleagues reported levels of wild-type mouse RPE phagosomes at ZT14 that were modestly but statistically significantly higher than off-peak phagosome counts but less than half of the number of RPE phagosomes seen at ZT1 [7]. Notably, both studies explored mice of the same genetic background, the C57BL/6J (C57) strain.

Our studies exploring mice of the 129T2/SvEmsJ (129) strain have not revealed elevated rod POS phagosome numbers outside the morning peak using similar methodology [10,11]. However, these studies quantified rod OS phagosome content of the RPE at ZT12 and ZT15 and not at ZT13.5 or ZT14. We wondered if a burst of POS phagocytosis after dark in 129 mice had been missed previously due to different sampling time points or other technical differences in experimentation among different laboratories such as facility lighting, antibody usage, and tissue preparation, which may confound data set comparison, as recently reviewed [4]. Alternately, 129 may differ in outer segment renewal rhythmicity from C57 mice inherently due to genetic differences. Indeed, numerous phenotypic, physiological, and behavioral differences exist among inbred mouse strains in general [12].

Here, we therefore directly compared RPE phagocytosis of both rod and cone POS in 129 and C57 mice raised under identical conditions with equal sample collection and processing at identical and relevant time points before and after light and dark onset. As the exposure of phosphatidylserine (PS) at the outer segment tip serves as “eat me” signal for the RPE and thus precedes phagocytosis by RPE [13], we additionally quantified PS exposure at rod outer segment tips in both 129 and C57 mice to further compare outer segment renewal in these widely used strains. Altogether, these experiments reveal principal similarity but also significant differences in the diurnal rhythm of outer segment renewal dependent on mouse strain.

## 2. Methods

### 2.1. Materials and Reagents

Materials were purchased from Thermofisher (Waltham, MA, USA) unless otherwise stated.

### 2.2. Animals

All procedures involving animals were pre-approved by Fordham University’s Animal Care and Use Committee (IACUC) and adhered to the ARVO Statement for the Use of Animals in Ophthalmic and Vision Research. Wild-type 129T2/SvEmsJ (129) and C57BL6/J (C57) mice originally purchased from Jackson Laboratories were bred, raised, and housed in the Fordham University animal facility under a strict 12 h light–dark cycle, with light onset at ZT0 and illuminance during the light period at cage-level of ~100 lux. Mice received standard rodent chow and water ad libitum. Experiments used mixed cohorts of male and female mice aged 5 months. Our previously published study demonstrated that there is no difference in morning POS phagocytosis between male and female 129 mice [14]. Cohort founder mice were tested and found to be negative for the rd8 mutation [15]. Mice were sacrificed by CO_2_ asphyxiation followed by cervical dislocation at specific time points relative to entrained light onset. Eye globes were enucleated, dissected, and processed immediately postmortem.

### 2.3. Whole-Mount Tissue Preparation, Immunofluorescence Labeling, and Microscopy

Eye globes were processed for whole-mount RPE rod and cone POS phagosome quantification exactly as described recently [16]. In brief, enucleated eyes were dissected in cold Hank’s buffered saline solution with calcium and magnesium (HBSS-CM, cat# 21-022CV, Corning Mediatech, Manassas, VA, USA) to remove the lens, cornea, iris, and neural retina. The resulting posterior eyecup was fixed for 30 min at room temperature in freshly prepared 4% paraformaldehyde in PBS followed by transfer to PBS. The eyecup was then cut radially to yield a flat, flower-like whole-mount sample with 4 petals and with the RPE facing up. Cuts were made from the edge toward the optic disc but did not cut into the optic disc. Whole mounts were incubated in a humidified chamber for the duration of immunofluorescence staining. They were blocked with 5% donkey serum, 1% BSA, and 0.3% Triton X-100 in PBS for 30 min at room temperature. Primary and secondary antibodies were diluted in 1% donkey serum, 1% BSA, and 0.1% Triton X-100 in PBS. Whole mounts were incubated with a mix of red–green cone opsin and blue cone opsin rabbit antibodies (cat#5405 and 5407, respectively, both Millipore-Sigma, St. Louis, MO, USA 1:300 each) overnight at 4 °C, washed with PBS, and incubated with donkey anti rabbit-AlexaFluor-594 antibody (cat# A-21207, Thermofisher, 1:250) at room temperature for 2 h. This was followed by incubation with primary mouse antibody to rhodopsin (clone B6-30, cat# NBP2-25160, Novus Biologicals, Centennial, CO, USA, 1:250) overnight at 4 °C, washing with PBS, and incubation with donkey anti mouse-AlexaFluor-488 antibody (cat# A-21202, Thermofisher, 1:250) at room temperature for 2 h. Whole mounts were mounted RPE side up on microscope slides using Vectashield (cat# H-1000, Vector Laboratories, Newark, CA, USA). Coverslips were sealed using nail polish. At least 8 x-y image stacks per whole-mount tissue were acquired using a Leica TSP8 confocal microscopy system. Image projections were recompiled in Adobe Photoshop 2021. Phagosomes defined as circular opsin signals of at least 0.5 μm in diameter were counted in maximal projections using ImageJ.

### 2.4. Live Imaging of Externalized PS at Outer Segment Tips

PS live imaging and analyses were performed exactly as reported previously [13,17]. In brief, neural retinae were dissected from eye globes in HBSS-CM at room temperature immediately following animal sacrifice. Both isolated, intact neural retinae from a mouse were transferred photoreceptor side up with a plastic transfer pipette cut obliquely to widen the opening onto a microscope slide prepared with hardened nail polish dots. Retinae were incubated in a 50 μL biosensor in HBSSCM (10 μg/mL, “polarity sensitive indicator of viability and apoptosis” pSIVA, cat# NBP2-29382, Novus Biologicals). A coverslip was placed on top of the nail polish dots with care not to crush retinae. Fluorescence signals indicating exposed PS were imaged within 30 min of mounting on a Leica TSP8 laser scanning confocal microscopy system. X-y image stacks were acquired and collapsed to yield maximal projections. These were recompiled in Adobe Photoshop 2021 for figures. Frequency of outer segment tips exposing PS per field and length of PS exposure of individual outer segment tips lab were quantified using ImageJ.

### 2.5. Statistical Analyses

Results were compiled, averaged, and analyzed statistically using GraphPad Prism. For each data point, at least 4 tissues from 4 different mice were analyzed. The exact tissue numbers for each experiment are provided in the figure legends. Quantification results were compiled, averaged, and analyzed statistically using GraphPad Prism. Two-way ANOVA testing with Tukey’s post hoc test was used for comparisons. Differences of *p* < 0.05 were considered statistically significant.

## 3. Results

### 3.1. Significantly Higher Numbers at ZT1 but No Peak after Dark in Rod POS Phagosome Content in 129 RPE Compared to C57 RPE

**Figure 1:**
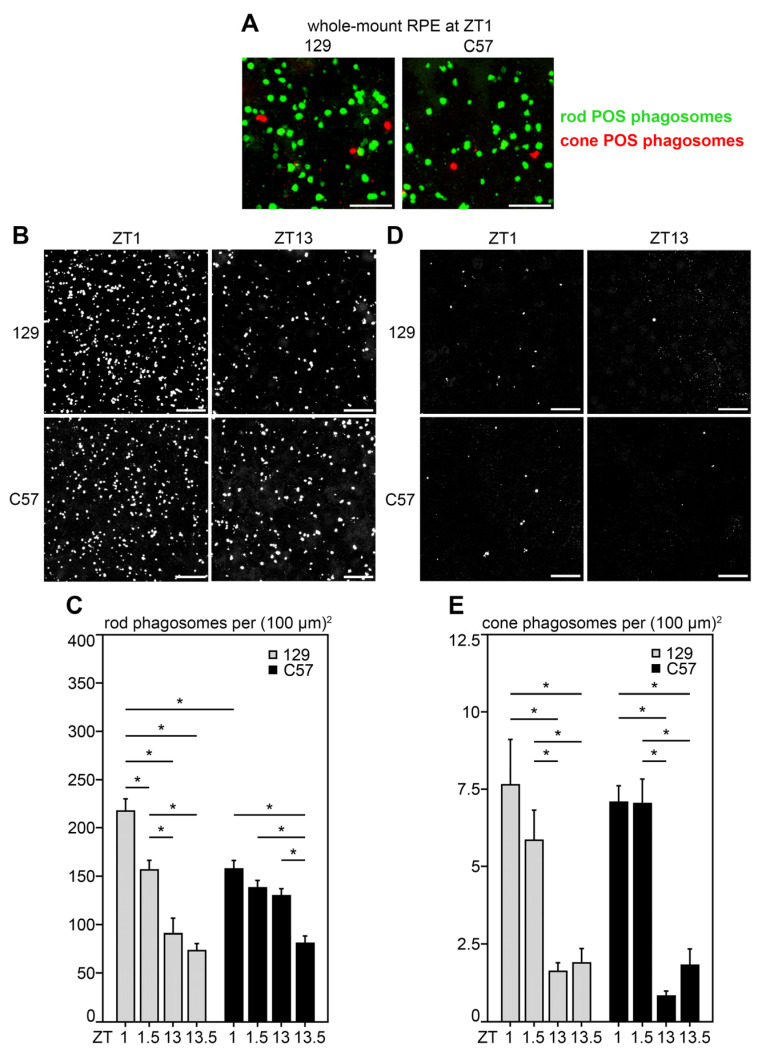
Differences in diurnal rod but not cone POS phagosome content in RPE of 129 and C57 mice: accentuated rod POS phagosome peak at ZT1 but no peak after dark in 129 RPE. RPE whole-mount tissues were collected at ZT1, ZT1.5, ZT13, and ZT13.5 followed by rod and cone phagosome labeling and fluorescence microscopy. (**A**) Representative high-magnification image stack maximal projections showing rod and cone POS phagosomes in green and red, respectively, at ZT1 in strains as indicated. Scale bars, 10 µm. (**B**) Representative low-magnification image stack maximal projections of rod POS phagosomes of 129 (**upper row**) and C57 (**lower row**) mice at ZT1 and ZT13 as indicated. (**C**) Quantification of rod POS phagosomes from images as shown in (**B**). (**D**) Representative image stack maximal projections of cone POS phagosomes of 129 (**upper row**) and C57 (**lower row**) mice at ZT1 and ZT13 as indicated. (**E**) Quantification of cone POS phagosomes from images as shown in (**D**). Scale bars in (**B**,**D**), 25 µm. Bars in (**C**,**E**) show mean ± s. e. m., *n* = 5 eyes from 5 different mice per sample; two-way ANOVA with Tukey’s post hoc test revealed significant differences between select sample groups as connected by lines with * indicating *p* < 0.05.

We prepared whole-mount RPE samples at specific times after light and dark onset from age-matched 129 and C57 mice that were born, raised, and light-entrained under the same conditions. We sought to ensure that we would be able to directly compare time points with earlier studies by us and others. As our previous studies focused on ZT1 and the recent data on C57 mice tested at ZT1.5, we collected tissues both 1 and 1.5 h after light and after dark, respectively (ZT1, ZT1.5, ZT13, and ZT13.5). We co-stained RPE whole mounts with rhodopsin and cone opsin antibodies to allow for identification of both rod and cone POS phagosomes by immunofluorescence microscopy. Close-up observation of samples obtained at ZT1 and co-stained for rod and cone POS phagosomes, respectively, did not reveal obvious differences in overall size, appearance, or distribution of phagosomes (Figure 1A). To quantify phagosomes, we next imaged and analyzed low-magnification fields in order to represent a large portion of the RPE in the analyses. Representative images for rod phagosomes (Figure 1B) and resulting quantification revealed that rod POS phagocytosis peaks at ZT1 in 129 mice with significantly fewer rod POS phagosomes at ZT1.5 (Figure 1C, gray bars). At both time points after dark, we found significantly lower rod POS phagosome content in 129 mice than at either ZT1 or ZT1.5 (Figure 1C, gray bars). There was no difference in rod POS phagosome content at ZT13 and ZT13.5 in 129 mice. In C57 mice, rod POS phagosome content was the same at ZT1, ZT1.5, and ZT13 and significantly less than any of these only at ZT13.5 (Figure 1C, black bars). Unlike in 129 mice, rod POS phagosome content at ZT1 was not significantly different from ZT1.5 in C57 mice. Comparing rod phagosome content between strains at the same time point, the rod POS phagosome content at ZT1 was significantly higher in 129 than in C57 mice (Figure 1C, gray and black bars, ZT1). Next, we quantified cone POS phagosome content in the RPE at the same time points. We found no difference between 129 and C57 RPE in cone POS phagosomes regardless of the time point tested (Figure 1D,E). Cone POS phagosome content was elevated in both strains shortly after light onset, at ZT1 and ZT1.5, as compared to shortly after dark, and there was no difference between ZT1 and ZT1.5 or ZT13 and ZT13.5, respectively (Figure 1D,E). Finally, it should be noted that the morphology of the RPE as well as the distribution of rod and cone POS phagosomes across retinal regions was indistinguishable between the two strains.

### 3.2. Attenuated Frequency of Photoreceptor Outer Segments Exposing PS at Their Distal Tip in C57 Compared to 129 Mice

**Figure 2:**
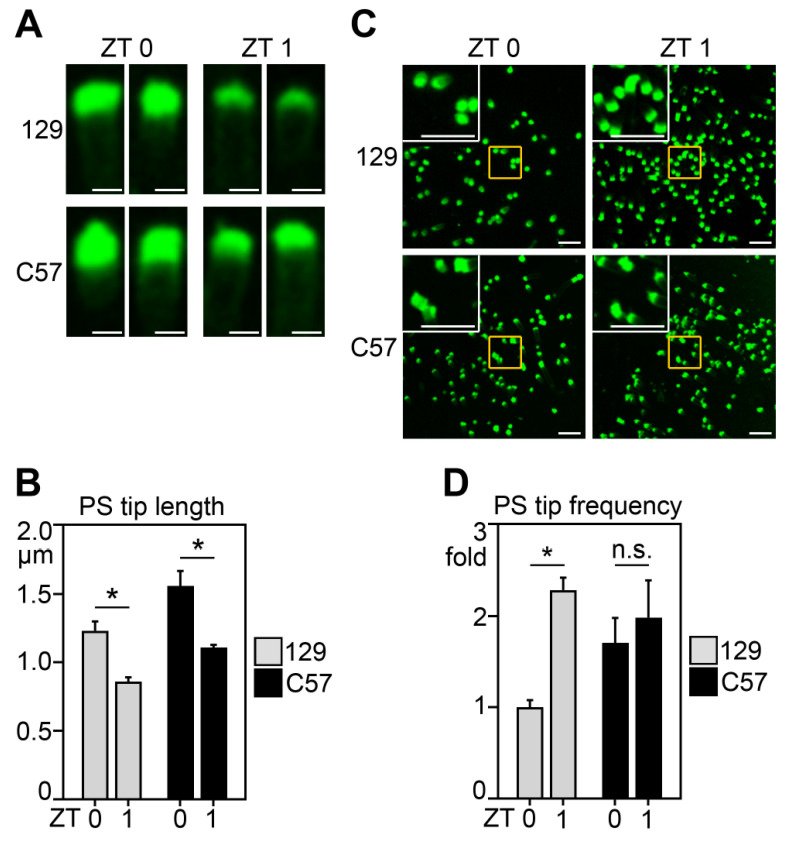
Similar diurnal elongation of PS exposure at rod outer segment tips but reduced PS-POS tip frequency in C57 compared to 129 mice. Whole-mount neural retina mounted outer segment side up was incubated with biosensor labeling externalized PS and live-imaged. (**A**) Images are representative maximal projections showing individual rod outer segments live-imaged at high magnification. Scale bars, 2 µm. (**B**) Quantification of the length of PS-exposing tips of outer segments from images as in (**A**). (**C**) Representative retina overview fields of PS biosensor live imaging experiments. Images are maximal projections. Inset with white borders in top left corner of each field shows magnification of areas indicated by yellow rectangles. All scale bars in (**C**), 10 µm. (**D**) Quantification of the frequency of outer segments exposing PS in C57 and 129 mice of samples as in (**B**). Values are normalized to the frequency of outer segments exposing PS in 129 mice at ZT0. Bars in (**B**,**D**) show mean ± s. e. m., *n* = 4–5 for (**B**), and *n* = 4 for (**D**); two-way ANOVA with Tukey’s post hoc test showed significant differences as indicated by lines (* indicates *p* < 0.05).

The rod POS phagosome content analyses above suggest an attenuated burst of RPE phagocytic activity toward rod POS after morning light onset in C57 mice compared to 129 mice. We wondered if this was reflected in differences in exposure of the PS “eat me” signal exposed by photoreceptor tips, which precedes the morning peak of RPE phagocytosis [13]. Previously, we showed that in 129 mice, PS tips elongate by ZT0 and show maximal frequency across the retina at ZT1 [13,17]. Here, we thus directly compared the length of PS-marked outer segment tips and frequency of PS-exposing outer segment tips in both 129 and C57 retina in the morning at ZT0 and ZT1. We found that the average length of PS exposure at individual outer segments was significantly higher at ZT0 than at ZT1 in both 129 and C57 retina, matching our previous studies (Figure 2A,B, compare ZT0 and ZT1 gray bars and black bars, respectively). Thus, individual rod photoreceptors in C57 retina respond to anticipated light onset like those in 129 retina, by elongating their PS-exposing tips. Moreover, PS-exposing outer segment tips were significantly longer at ZT1 in C57 than in 129 retina (Figure 2B, compare gray to black bars at ZT1 and at ZT1.5, respectively). With respect to frequency of rods with PS tips exposing PS, we imaged low-magnification fields (Figure 2C). Insets with white borders in the upper left-hand corner of each panel show enlargements of areas indicated by yellow squares in each field. These illustrate that overall, the appearance of PS tip labeling was similar among strains. Quantification showed that the abundance of PS-exposing outer segment tips in 129 retina more than doubled between ZT0 and ZT1, in agreement with our previous studies (Figure 2C,D, gray bars). In contrast, there was no change in frequency of rods with PS-exposing tips in C57 retina in this time period (Figure 2C,D, black bars). The average frequency of PS-exposing outer segment tips in C57 retina was in between the low average at ZT0 and the high average at ZT1 in 129 mice, but differences between strains did not reach statistical significance.

## 4. Discussion

In this brief report, direct comparison in side-by-side experiments supports the conclusion of significant differences in the diurnal rhythm of outer segment renewal between two widely used wild-type mouse strains, C57 and 129 mice. Differences were modest in size, and they centered on attenuated activity after light onset in C57 mice and the lack of a burst in rod POS phagocytosis after dark onset in 129 mice. These data underscore the importance of selection, control, and precise reporting of genetic background strains as well as precise sampling times for studies of outer segment renewal in mice.

Our analyses of the whole-mount RPE labeled with rhodopsin antibody showed 1.6-fold higher peak rod POS phagosome numbers in 129 than in C57 mice at ZT1. Moreover, rod POS phagosome content in 129 RPE was 1.7-fold higher at ZT1 than at ZT1.5, and 2.4 and 3.2-fold higher than at ZT13 or ZT13.5, respectively. In contrast, rod POS phagosome content in the C57 RPE was similar at ZT1, ZT1.5, and ZT13 but lower at ZT13.5. These results corroborate the recently reported increase in rod POS phagocytosis after dark onset in C57 mice but fail to detect a similar burst in 129 mice. Notably, the peak in rod POS phagocytosis at ZT1 in 129 mice was significantly greater than ZT1.5 in 129 mice. Thus, the daily peak of POS phagocytosis after light onset will be missed entirely if samples are collected only at ZT1.5. Moreover, it is critically important to be aware that relatively small changes in sampling time between ZT1 and ZT1.5 will greatly affect rod POS phagosome quantification. Sampling precisely at ZT1 will facilitate comparison of peak rod POS phagocytosis in future studies comparing outer segment renewal among wild-type and mutant mice.

Outer segment renewal involves synchronized collaboration of photoreceptors and RPE cells. PS exposure by photoreceptor outer segments precedes and is a prerequisite for POS phagocytosis. Exposure by the rod outer segment of PS is not cell-autonomous. Modifications in RPE receptors or RPE ligand recognition have been shown to alter PS exposure by outer segments although the missing proteins are not expressed by rods themselves [13]. Here, we found that lower rod POS phagosome content at ZT1 was matched by reduced frequency of PS-exposing outer segments in the retina of C57 mice. Notably, the extent of PS exposure by individual rod outer segments was at least as robust in C57 retina as in 129 retina, and in both strains, elongation of PS exposure along outer segments decreased within 1 h after light onset (implying removal by phagocytosis of the PS-exposing tip). Indeed, we did not observe principally different qualities or obvious differences in rod POS phagosome size, distribution, or in appearance or distribution of PS-exposing tips at any time point tested. Altogether, our data on PS exposure fully support the interpretation of the rod POS phagosome quantification: compared to 129 retina, rod POS turnover in C57 retina was attenuated after light onset but shows a second peak of activity after dark onset as recently shown by others.

Earlier studies did not specifically investigate the diurnal rhythm of content of cone-opsin-positive phagosomes in the RPE. However, we and others previously used commercially available polyclonal antibodies to identify cone POS phagosomes in the RPE [16,18]. Here, we used this well-established methodology to label cone POS phagosome in the same whole-mount samples used for rod POS phagosome quantification. Interestingly, we did not find a difference in cone POS phagosome content between C57 and 129 mice. In both mouse strains, cone POS phagosome numbers in the RPE were similar at ZT1 and ZT1.5, and these morning numbers were about 4-fold higher than at similar times after dark. While absolute numbers of cone POS phagosomes are necessarily low due to the much lower abundance of cones than of rods, our data do not support a burst of cone POS phagocytosis after dark onset in either C57 or 129 mice.

Our analysis of outer segment PS exposure in both strains showed that, after light onset, fewer outer segments exposed PS in C57 than in 129 retina, and this resulted in fewer rod POS phagocytosed by C57 RPE. The specific genetic differences in 129 mice underlying the attenuated morning phagocytic peak in C57 RPE are not known at this time and will likely require considerable effort to identify as genetic differences among these two and other laboratory mouse strains are extensive [19].

C57 and 129 mice have been shown to differ in the RPE phagocytic machinery itself, namely in expression of the receptor tyrosine kinase TYRO3, which may substitute for the related MERTK in promoting POS engulfment by RPE cells [20]. However, the moderate differences in morning peak size are unlikely to be caused by such differences in phagocytic receptor expression. Signaling by the clock regulatory hormone melatonin has been shown to play a role in the timing of the morning RPE phagocytic peak: knocking out melatonin receptor type 2 but not type 1 in melatonin-proficient C3H-f mice caused a phase advance in the peak of RPE phagocytosis [21]. Most commonly studied inbred mouse strains, including C57- and 129-related strains, however, produce no or very low levels of melatonin systemically and in the retina [22,23]. Earlier studies have revealed genetic causes [24,25] and physiological consequences of insufficiency in melatonin of C57 mice specifically (recently reviewed in [26]). Published studies are in overall agreement that melatonin insufficiency may dampen rhythmic aspects of tissue physiology but does not eliminate circadian processes including, notably, circadian changes in the RPE transcriptome [27]. Further directly relevant to the present study, administration of exogenous melatonin does not increase RPE phagocytosis at presumptive light onset when C57 mice are housed in constant darkness, although it rescues circadian dopamine synthesis [28]. Much evidence, however, implicates dopamine in circadian regulation in C57 retina [29]. Indeed, C57 mice constitutively lacking expression of dopamine receptor 2 show constant levels of rod POS phagosome numbers in the RPE at all sampling times including ZT1 and ZT14 [7]. Future studies will be needed to assess whether differences in regulation of dopamine or its downstream signaling are directly relevant to the enhanced peak of rod POS turnover in 129 mice. With respect to genes/proteins directly involved in circadian regulation, RPE-specific knockdown of the essential clock gene *Bmal1* was sufficient to abolish rhythmic phagocytosis, while retina-specific knockdown of *Bmal1* had no effect on phagocytosis [5]. To our knowledge, 129 mice have not yet been studied specifically with respect to circadian regulation in the retina. However, a recent study demonstrated in adrenal glands and liver that the expression of core clock genes differs between C57 mice and 129/S2 mice [30]. Interestingly, this study found generally lower amplitudes of clock gene expression in C57 mice and differences in expression of clock genes including, intriguingly, *Bmal1* [30]. Other studies showed differences in C57 mice compared to 129-type sub-strains in free-running period, daily activity, and food intake [31,32,33]. It should be noted that the numerous available 129 sub-strains also significantly differ from each other in circadian activity [32]. Thus, additional studies are needed focusing specifically on the sub-strain we explored, 129T2/SvEmsJ. Surely, direct comparison of circadian activity monitoring and gene expression in the RPE and neural retina between 129T2/SvEmsJ and C57 mice would be informative for future research, but these extensive tests are beyond the scope of this brief report.

Altogether, our work shows strain-specific differences in the rhythm of rod outer segment renewal between C57 and 129 mice. Albeit subtle, these changes are significant in that they may complicate comparisons across strains. Whether or not the underlying molecular mechanisms will be identified, knowledge of the specific time course of outer segment renewal in the two widely used mouse strains explored here needs to be considered when selecting sampling time points in future investigations.

## Data Availability

Experimental data are available from the corresponding author upon request.
